# Quantitative salivary gland SPECT/CT using deep convolutional neural networks

**DOI:** 10.1038/s41598-021-87497-0

**Published:** 2021-04-09

**Authors:** Junyoung Park, Jae Sung Lee, Dongkyu Oh, Hyun Gee Ryoo, Jeong Hee Han, Won Woo Lee

**Affiliations:** 1 Department of Biomedical Sciences, Seoul National University College of Medicine, 103 Daehak-ro, Jongno-gu, Seoul, 03080 Korea; 2 Department of Nuclear Medicine, Seoul National University College of Medicine, 103 Daehak-ro, Jongno-gu, Seoul, 03080 Korea; 3 Department of Nuclear Medicine, Seoul National University Bundang Hospital, 82, Gumi-ro 173 Beon-gil, Bundang-gu, Seongnam-si, 13620 Gyeonggi-do Korea; 4 Institute of Radiation Medicine, Medical Research Center, Seoul National University, Seoul, Korea

**Keywords:** Oral diseases, Diagnostic markers, Translational research

## Abstract

Quantitative single-photon emission computed tomography/computed tomography (SPECT/CT) using Tc-99m pertechnetate aids in evaluating salivary gland function. However, gland segmentation and quantitation of gland uptake is challenging. We develop a salivary gland SPECT/CT with automated segmentation using a deep convolutional neural network (CNN). The protocol comprises SPECT/CT at 20 min, sialagogue stimulation, and SPECT at 40 min post-injection of Tc-99m pertechnetate (555 MBq). The 40-min SPECT was reconstructed using the 20-min CT after misregistration correction. Manual salivary gland segmentation for %injected dose (%ID) by human experts proved highly reproducible, but took 15 min per scan. An automatic salivary segmentation method was developed using a modified 3D U-Net for end-to-end learning from the human experts (*n* = 333). The automatic segmentation performed comparably with human experts in voxel-wise comparison (mean Dice similarity coefficient of 0.81 for parotid and 0.79 for submandibular, respectively) and gland %ID correlation (*R*^2^ = 0.93 parotid, *R*^2^ = 0.95 submandibular) with an operating time less than 1 min. The algorithm generated results that were comparable to the reference data. In conclusion, with the aid of a CNN, we developed a quantitative salivary gland SPECT/CT protocol feasible for clinical applications. The method saves analysis time and manual effort while reducing patients’ radiation exposure.

## Introduction

Salivary glands are multifunctional organs with protective, digestive, exocrine, and endocrine functions. Dysfunction of the glands poses serious problems in a variety of medical situations. For example, thyroid cancer patients often experience sialadenitis after radioactive iodine (RAI) therapy^1^, and reduced parotid function is considered the hallmark RAI-induced adverse effect. Autoimmune causes of sialadenitis, such as Sjögren’s syndrome, show severely impaired uptake/excretion of entire salivary glands in the chronic stage^2^, whereas excretory dysfunction of the submandibular glands appears in the early stage of the disease^3^. Recently, xerostomia has emerged as one of main side effects of alpha-particle-based radionuclide therapy for advanced prostate cancer^4^.

The function of salivary glands has been evaluated using Tc-99m pertechnetate because it follows the natural process of saliva production and excretion^3, 5–9^. However, the protocol of salivary gland scintigraphy has not yet been standardized: multiple static planar scans^3, 5, 9^ or dynamic planar scans with either time-activity-curve^6, 7^ or uptake/excretion pattern analysis^8^ have been tested, but no consensus has been reached by the major nuclear medicine societies. Thus, there is an unmet need for proper salivary gland scintigraphy using Tc-99m pertechnetate^10, 11^.

Quantitative single-photon emission computed tomography/computed tomography (SPECT/CT) is a promising method for the functional evaluation of organs^12^. Recent technological development has allowed the application of quantitative nuclear imaging parameters, such as %injected dose (%ID) and standardized uptake value (SUV), to SPECT/CT imaging^13–21^. Quantitative salivary gland SPECT/CT holds promise as a new imaging modality^12, 22^ since the interpretation of the salivary gland scintigraphy depends on the quantitation of the uptake (i.e., %ID) of Tc-99m pertechnetate by the salivary glands. However, some issues remain to be solved before salivary gland SPECT/CT can be widely applied. First, salivary gland segmentation is not an easy task because the parotid and submandibular glands are not well visualized on CT used for segmentation during the salivary gland SPECT/CT. Second, previously developed CT-based automatic segmentation methods focused on the accurate identification of salivary glands that are at risk of external radiation therapy, lacking implications for nuclear medicine^23–25^. Lastly, the performance of human experts who trained the automatic segmentation algorithm has not been presented in previous studies^23, 25–27^. Therefore, it is not clear whether the reported automatic algorithms are of use in the real world of clinical practice^28^.

This study develops a novel salivary gland SPECT/CT protocol (pre-stimulation SPECT/CT and post-stimulation SPECT), in which salivary gland function is represented by the absolute quantitation of Tc-99m pertechnetate uptake (i.e., %ID). Using a deep learning approach, we replace manual segmentation with an automated segmentation method. Furthermore, the performance of human experts who trained the automatic algorithm is presented.

## Results

### Reproducibility of salivary gland manual segmentation by the human experts who trained the network

Inter-operator reproducibility was investigated by two nuclear medicine physicians (JHK and DGO), who were senior residents of nuclear medicine and had had more than 3 years of clinical experience and proficiency in salivary segmentation. The first investigator (JHK) produced significantly smaller VOIs for parotid glands (23.43 ± 10.42 mL, *p* = 0.0008 by a paired *t* test) but bigger VOIs for submandibular glands (11.57 ± 4.48 mL, *p* < 0.0001 by a paired *t* test) compared to the second investigator (DGO) (25.46 ± 10.70 mL for the parotid gland and 9.80 ± 3.71 mL for the submandibular gland). This tendency was directly reflected in the %ID: the parotid %ID of the first investigator (0.27 ± 0.13%) was significantly lower than that of the second investigator (0.30 ± 0.14%, *p* < 0.0001 by a paired *t* test), whereas the submandibular %ID of the first investigator (0.19 ± 0.11%) was significantly greater than that of the second investigator (0.16 ± 0.10%, *p* < 0.0001 by a paired t test). However, the inter-operator reproducibility, as represented by the ICC, between the two investigators was excellent for %ID and VOI size, whereas the %ID had greater ICC values than the VOI size for both the parotid and submandibular glands (Table 1). The Dice similarity coefficient (DSC) values between the two experts were 0.77 ± 0.04 for the parotid gland and 0.81 ± 0.08 for the submandibular gland.

**Table 1:** ICCs for %ID by SPECT and VOI size by CT (*n* = 30).

	%ID		VOI size	
	Parotid	Submandibular	Parotid	Submandibular
Inter-operator	0.9020 (0.7456–0.9537)	0.9595 (0.7414–0.9861)	0.8954 (0.7992–0.9424)	0.8415 (0.2660–0.9442)
Intra-operator	0.9541 (0.9243–0.9723)	0.9667 (0.9440–0.9803)	0.9260 (0.8791–0.9551)	0.8539 (0.7635–0.9116)

Parentheses indicate the 95% confidence interval of the ICC.

Intra-operator reproducibility was assessed by the third investigator (JHH), who was a pioneer in salivary SPECT/CT with more than 10 years of experience in nuclear medicine imaging analyses. No significant difference was observed between the investigator’s own first and second datasets of parotid VOIs (19.60 ± 8.60 mL vs. 19.45 ± 8.75 mL, *p* = 0.7279 by a paired *t* test). Accordingly, the parotid %ID did not differ significantly between the first and second datasets (0.25 ± 0.12% vs. 0.25 ± 0.12%, *p* = 1.000 by a paired *t* test). Similarly, for the submandibular gland VOIs, no significant difference was found between the first (9.74 ± 3.15 mL) and second (9.94 ± 3.47 mL, *p* = 0.4080 by a paired t-test) datasets. Consequently, submandibular %IDs were not significantly different between the first (0.16 ± 0.10%) and second (0.17 ± 0.10%, *p* = 0.5747 by a paired *t* test) datasets. The ICC values for intra-operator reproducibility were also excellent, and the %ID again had higher ICCs than the VOI size for both the parotid and submandibular glands (Table 1). The DSC values for the two datasets were 0.84 ± 0.05 for the parotid gland and 0.84 ± 0.04 for the submandibular gland.

As expected, ICCs for intra-operator reproducibility were always greater than those for inter-operator reproducibility under all conditions, whether for glands (parotid or submandibular) or parameters (%ID or VOI size) (Table 1).

### Effects of misregistration correction between the 20-min CT and 40-min SPECT

**Figure 1:**
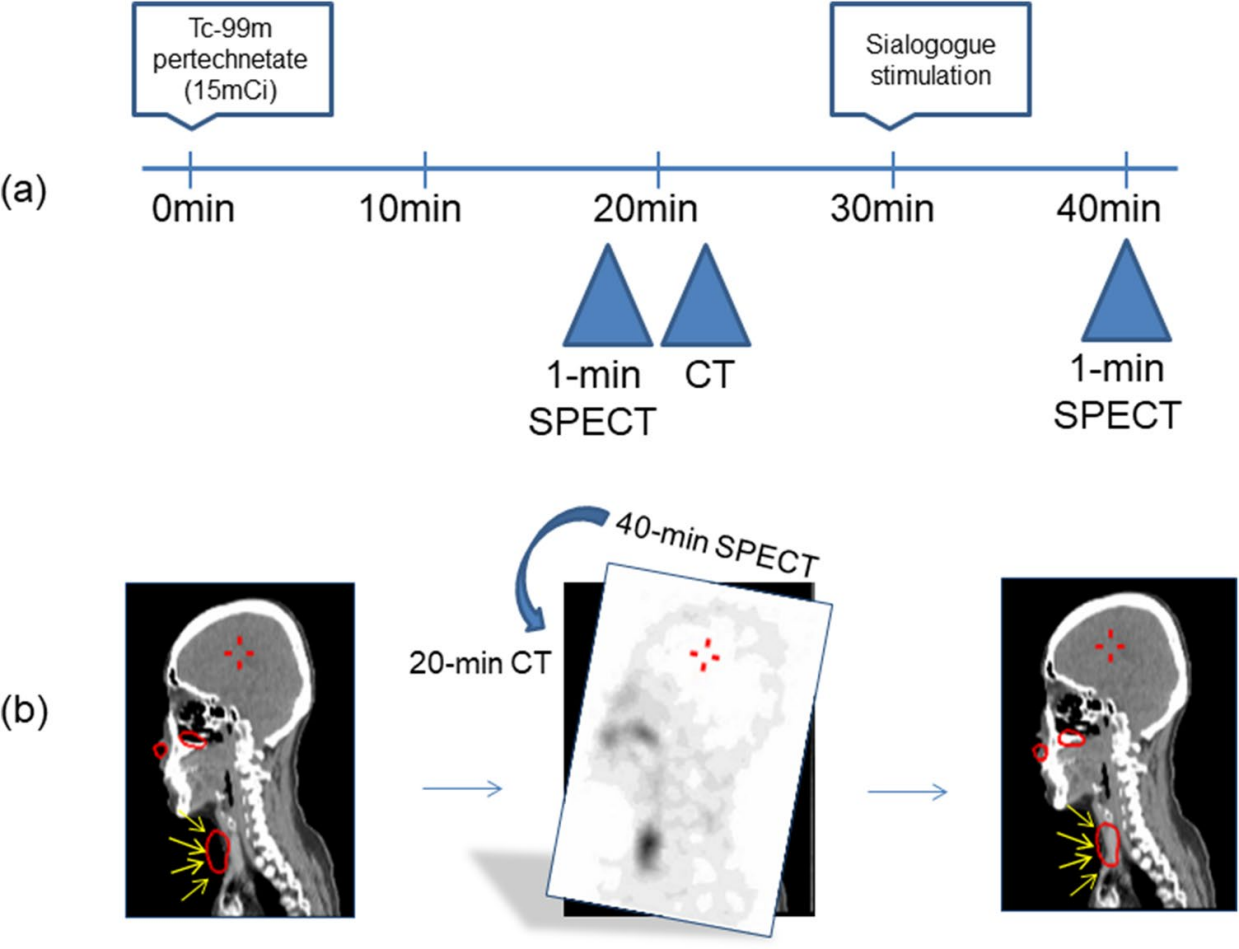
(**a**) Proposed salivary gland SPECT/CT protocol. (**b**) A 20-min CT was employed to reconstruct the 40-min SPECT; misregistration between the CT and SPECT was corrected using vendor-provided quality control functions (Hybrid QC, Preparation for Q.Metrix, GE). The misregistration correction process is three-dimensional in nature, but only sagittal plane images are presented for convenience. Please note that misregistered thyroid activity (the large ROI in red) is correctly adjusted to the genuine thyroid tissue after the quality control process (yellow arrows).

Thirty-eight parotid and 34 submandibular glands in 19 patients were investigated by one investigator (DGO). In most cases, misregistration between the 20-min CT and 40-min SPECT was insignificant, but in some instances, the position difference between the two sets of SPECT/CTs was substantial^12^. When misregistration between the 20-min CT and the 40-min SPECT was corrected (Fig. 1b), the 40-min SPECT %IDs, which had been significantly biased in some cases, became comparable to the reference data (Supplementary Fig. 3). We therefore concluded that the 40-min CT might not be essential in the proposed salivary gland SPECT/CT protocol (Fig. 1). We applied this protocol to the automatic segmentation algorithm development and its verification. The details of the effects of misregistration correction are presented in the “Supplementary material S1”.

### Development of the automatic segmentation algorithm

**Figure 2:**
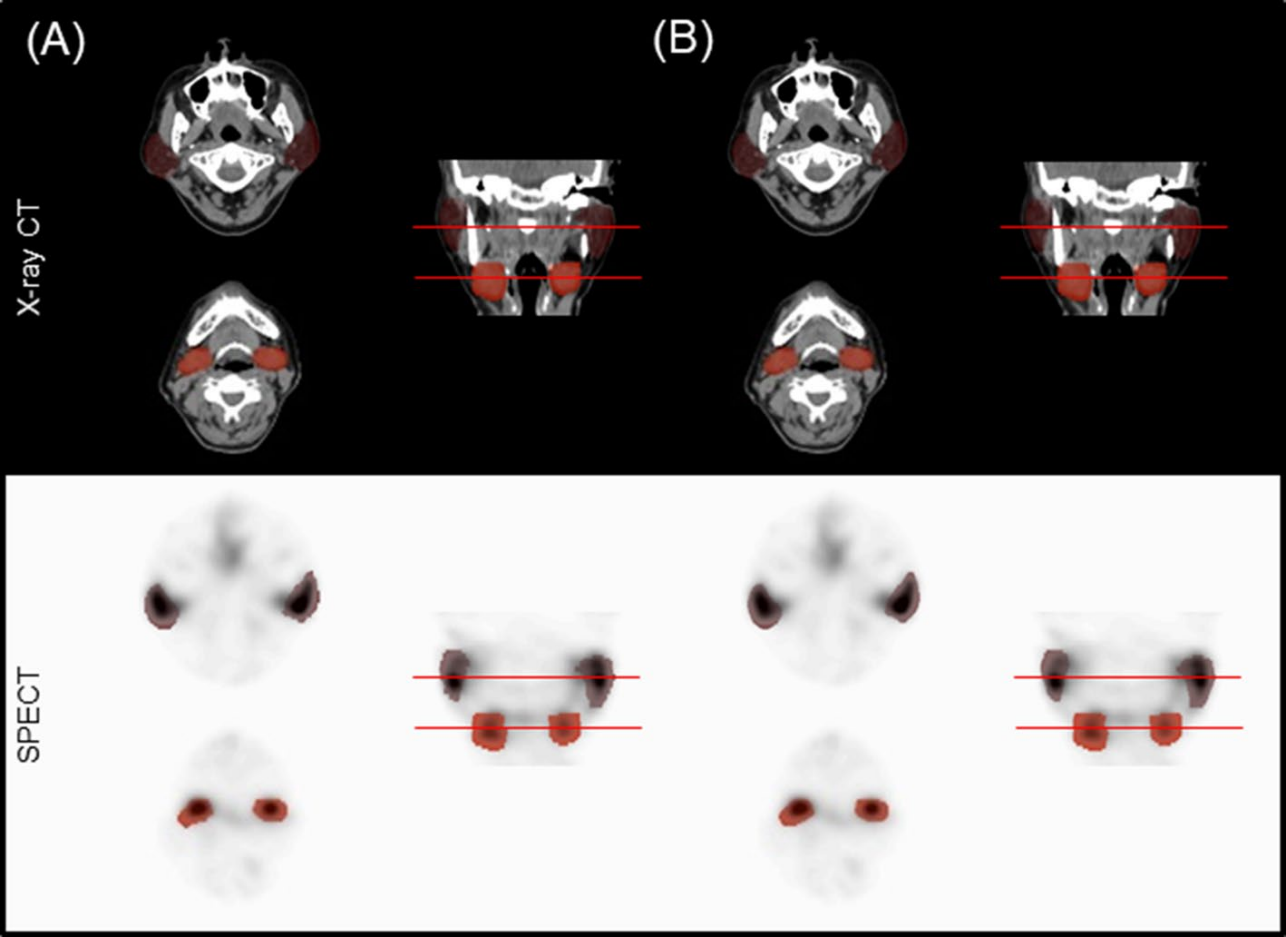
Salivary gland segmentation results. Representation of axial images, with the red bar in the coronal images indicating the ROIs for the parotid (upper image) and submandibular (lower image). (**a**) Manually segmented ROI. (**b**) Deep-learning-generated automatic ROI.

We successfully developed an automatic salivary gland segmentation algorithm using 20-min CT images. The manual and automatic segmentation methods performed comparably. Results obtained from both cases included high activity of the parotid and submandibular glands in SPECT images (Fig. 2). For the parotid gland %ID, the algorithm achieved a high Dice similarity coefficient (DSC) relative to that of manual segmentation (mean ± SD = 0.81 ± 0.09 for the main experiment in Table 2). The mean absolute percentage difference for the parotid %ID between the two methods was found to be 7.75 ± 8.28%. Cross-validation yielded the following values: 9.46 ± 10.41%, 8.21 ± 8.18%, 8.59 ± 7.08%, and 10.03 ± 10.74%. Correlation coefficient *R*^2^ consistently ranged from 0.93 to 0.94. Nearly identical results (i.e., a high DSC of 0.79 ± 0.09, low mean absolute percentage difference of 10.43 ± 12.02%, and high correlation of 0.95 between manual and automatic segmentation) were obtained for the submandibular glands (Table 3).

**Table 2:** Cross-validation results for parotid %ID.

Method	Unit	Dataset				
		Main experiment	Cross-validation 1	Cross-validation 2	Cross-validation 3	Cross-validation 4
DSC	(mean ± SD)	0.81 ± 0.09	0.81 ± 0.07	0.80 ± 0.07	0.80 ± 0.07	0.79 ± 0.07
	[range]	0.57–0.96	0.62–0.96	0.63–0.95	0.63–0.96	0.56–0.95
Mean-M	% (mean ± SD)	0.35 ± 0.22	0.31 ± 0.14	0.37 ± 0.18	0.33 ± 0.14	0.33 ± 0.17
Mean-A	% (mean ± SD)	0.33 ± 0.17	0.32 ± 0.12	0.36 ± 0.16	0.33 ± 0.14	0.33 ± 0.15
Correlation	*R*^2^	0.93	0.94	0.93	0.93	0.94
MAPE	% (mean ± SD)	7.75 ± 8.28	9.46 ± 10.41	8.21 ± 8.18	8.59 ± 7.08	10.03 ± 10.74

*DSC* dice similarity coefficient, *M* manual segmentation, *A* automatic segmentation, *MAPE* mean absolute percentage error.

**Table 3:** Cross-validation results for submandibular %ID.

Method	Unit	Dataset				
		Main experiment	Cross-validation 1	Cross-validation 2	Cross-validation 3	Cross-validation 4
DSC	(mean ± SD)	0.79 ± 0.09	0.81 ± 0.07	0.80 ± 0.07	0.80 ± 0.07	0.79 ± 0.07
	[range]	0.57–0.96	0.62–0.96	0.63–0.95	0.63–0.96	0.56–0.95
Mean-M	% (mean ± SD)	0.18 ± 0.12	0.15 ± 0.09	0.17 ± 0.09	0.17 ± 0.09	0.16 ± 0.08
Mean-A	% (mean ± SD)	0.16 ± 0.09	0.15 ± 0.08	0.17 ± 0.08	0.16 ± 0.09	0.16 ± 0.08
Correlation	*R*^2^	0.95	0.95	0.92	0.97	0.96
MAPE	% (mean ± SD)	10.43 ± 12.02	11.01 ± 10.35	11.79 ± 11.59	7.86 ± 6.85	8.55 ± 8.47

*DSC* dice similarity coefficient, *M* manual segmentation, *A* automatic segmentation, *MAPE* mean absolute percentage error.

**Figure 3:**
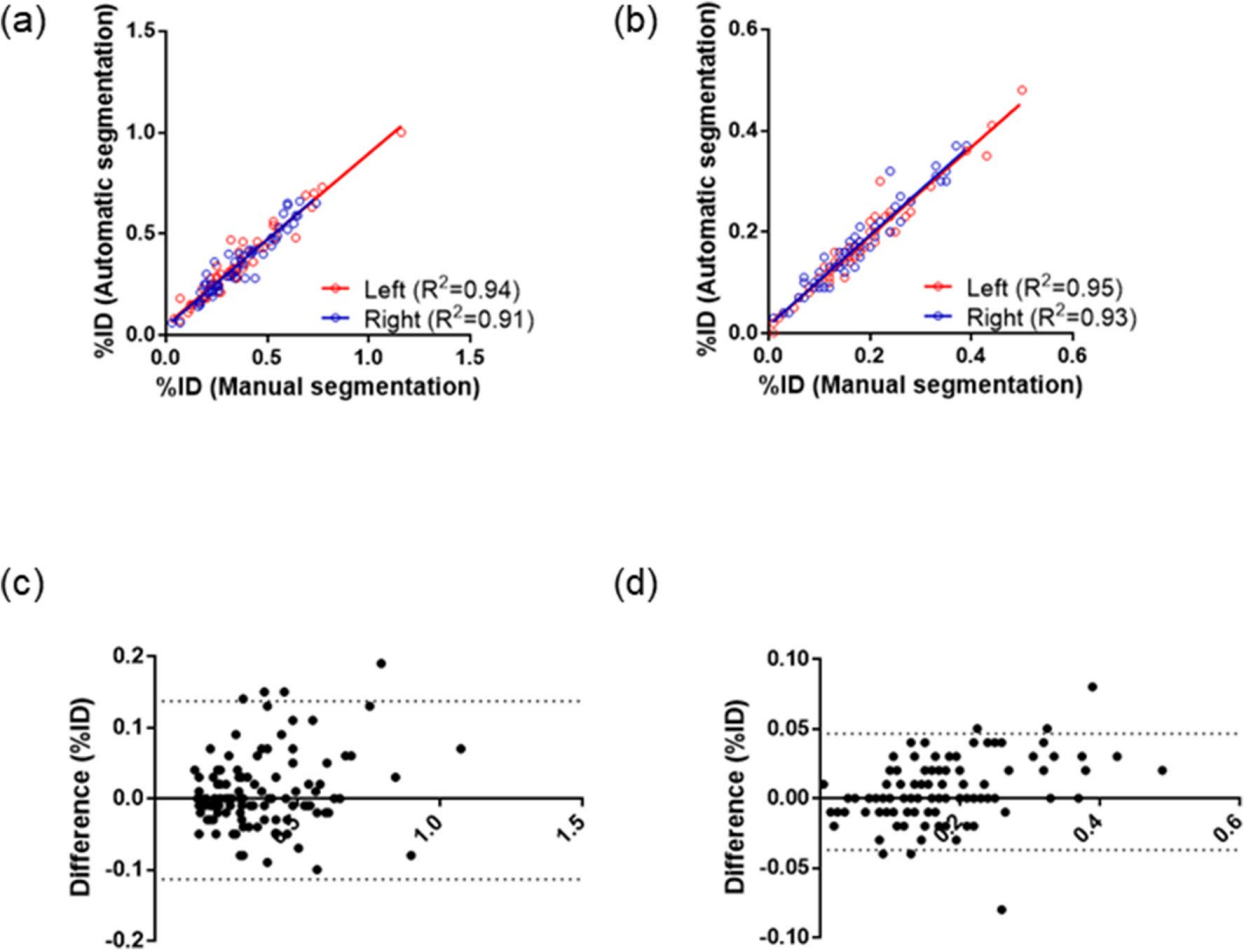
(**a**, **b**) Scatter plots and (**c**, **d**) Bland–Altman analyses of %ID measurements, using manual and deep-learning-generated volumes for the (**a**, **c**) parotid and (**b**, **d**) submandibular glands in the main experiment.

The high correlations (*R*^2^ = 0.93 for the parotid glands and *R*^2^ = 0.95 for the submandibular glands) and the minimal bias (absolute difference of 0.02% for each of the parotid and submandibular glands) of the %ID values between manual and automatic segmentation methods were demonstrated for the main experiment in Fig. 3. Supplementary Fig. 4 shows the mean absolute percentage error of %ID between the measurements obtained using manual and CNN-based volumes in fivefold cross-validation.

### Elapsed time of the automatic segmentation

The calculation of %ID and %EF took less than 1 min using the automatic algorithm with a given image dataset of SPECT and CT. The total elapsed time consisted of preprocessing (< 1 s), loading the network model (12 s), running the model (45 s), and post-processing (< 1 s). Compared to at least 15 min for manual salivary segmentation, the automated segmentation algorithm saves significant human resources.

### Performance of the automatic segmentation algorithm

The performance of the developed automatic algorithm was compared with that of a trainee (HGR). The segmentation results (%ID and %EF) of the apparently normal salivary glands compiled by the trainee and the automatic methods were compared with 43 reference normal data from our previous SPECT/CT report^12^.

**Figure 4:**
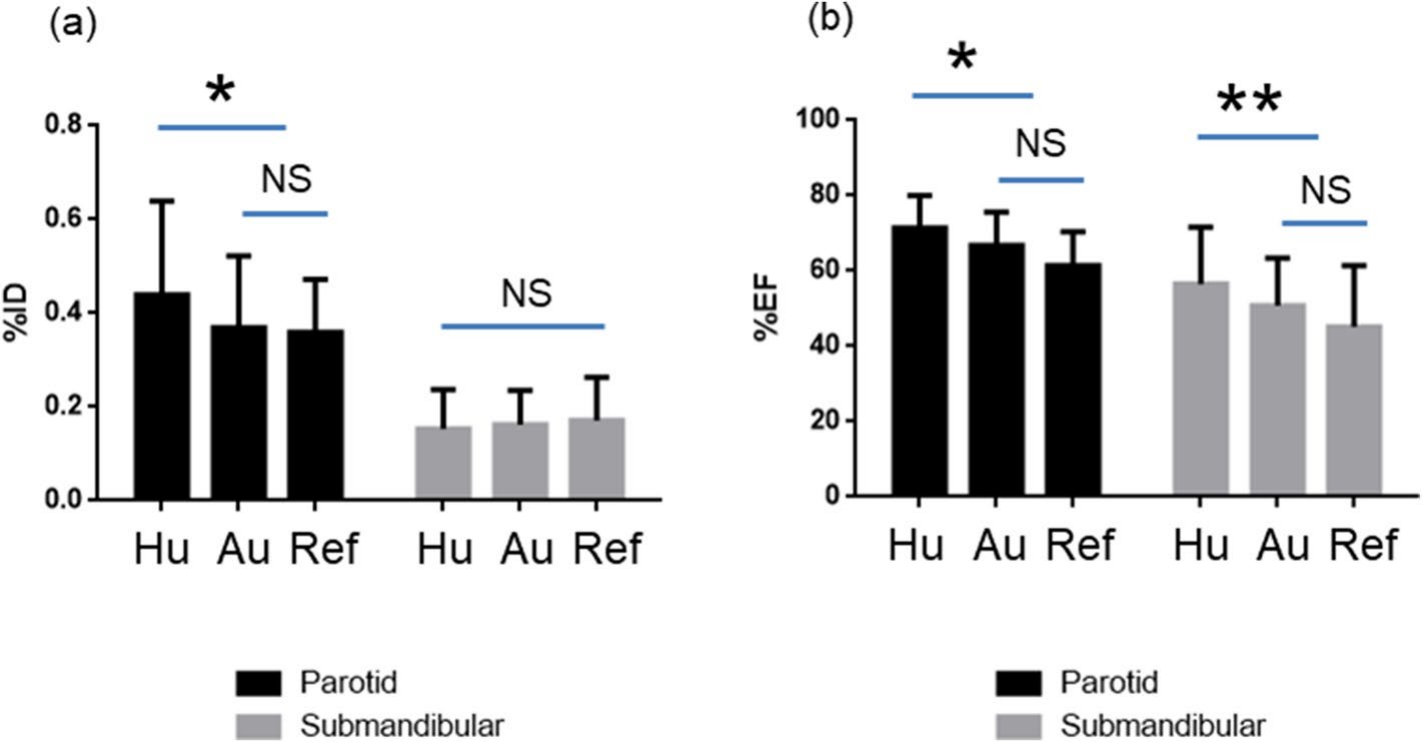
Comparison of segmentation results in normal salivary glands regarding the (**a**) %ID and (**b**) %EF between a human beginner (Hu) and the automatic segmentation algorithm (Au) in comparison with the reference results (Ref). The trainee generated significantly greater parotid %ID (*p < 0.0001), parotid %EF (*p < 0.0001), and submandibular %EF (**p = 0.0003), but the automatic algorithm produced equivalent %ID and %EF in both the parotid and submandibular glands (*NS* not significant).

The trainee tended to produce significantly higher values for the parotid %ID (0.44 ± 0.20%, *p* < 0.0001) (Fig. 4a), parotid %EF (71.41 ± 8.63%, *p* < 0.0001), and submandibular %EF (56.55 ± 15.10%, *p* = 0.0003) (Fig. 4b) than the automatic algorithm and the reference. The algorithm-generated %ID values (0.37 ± 0.15% and 0.16 ± 0.07% for the parotid and submandibular glands, respectively) were similar to the reference %ID values (0.36 ± 0.11% and 0.17 ± 0.09% for the parotid and submandibular glands, respectively) without a statistically significant difference (Fig. 4a). The developed algorithm produced comparable %EF values (66.83 ± 8.89% and 50.77 ± 12.68% for the parotid and submandibular glands, respectively) with the reference %EF (61.41 ± 9.04% and 45.22 ± 16.14% for the parotid and submandibular glands, respectively) and no statistically significant difference was observed (Fig. 4b).

### Radiation exposure to patients

The original salivary SPECT/CT had had an effective dose of 7.59 mSv (7.22 mSv by 555 MBq of Tc-99m pertechnetate plus 0.37 mSv by two CT sessions)^12, 29^. The single CT session elicited a CTDI_vol_ of 1.49 mGy and produced a dose-length-product of 60.31 mGy-cm in the X-ray conditions, resulting in a CT effective dose of 0.19 mSv. Thus, the omission of one CT session in the proposed salivary SPECT/CT protocol reduced the total effective dose to 7.41 mSv.

## Discussion

Because salivary dysfunction has emerged as the major adverse effect of theranostic cancer treatment, and especially of alpha-particle therapy^3, 4, 9, 30^, the need for functional imaging studies of the salivary glands has increased drastically. Traditional nuclear imaging of Tc-99m pertechnetate salivary scintigraphy has advantages and disadvantages^3, 5–9^, but it is uncertain whether salivary gland scintigraphy has played a proper role in salivary gland function evaluation, mainly because of poor reproducibility and lack of objectivity^5, 31^. Consequently, other nonnuclear imaging studies such as ultrasonography, CT, or MRI are emerging as alternatives to scintigraphy for a variety of salivary gland diseases^32–35^. However, because functional rather than anatomic deterioration is more important in cases of radionuclide therapy, more reliable and objective methods of salivary gland imaging are required.

There are reasons to advocate the proposed novel salivary gland SPECT/CT protocol (Fig. 1). First, it is an accurate quantitative tool for evaluating salivary gland function. The statement is based on the recent progress in quantitative SPECT/CT technique^22^, which has been proven in a variety of clinical applications^13–21^. Notably, the current SPECT/CT protocol produced normal %ID (0.36 ± 0.02% for the parotid and 0.17 ± 0.01% for the submandibular) and %EF (61.41 ± 1.38% for the parotid and 45.22 ± 2.46% for submandibular)^12^ reference ranges, which are comparable to the results obtained from conventional planar imaging^36, 37^. Furthermore, the DSC value obtained in the current study (0.81 ± 0.09) was comparable to the best reported result (0.81 ± 0.08 for the parotid gland) derived from the sophisticated two-step deep learning approach^38^. Accurate segmentation of the submandibular glands has not been reported previously, except in the current study. Second, patient radiation exposure reasonably decreases through the employment of only one CT session. The technique of misregistration correction between SPECT and CT may be extended to more than two SPECT/CT sessions, which means that single SPECT/CT and other multiple SPECTs can be considered equal to multiple sets of complete SPECT/CT images, reducing CT-induced radiation exposure to patients. SPECT acquisition lasted only 1 min in the current protocol. Thus, more than 10 quantitative SPECTs before and after stimulation may be realized with the aid of a single CT, enabling dynamic evaluation of salivary gland function, which has never been tested before. Lastly, the %ID and the %EF as imaging biomarkers for salivary function are analyzed in a consistent way, owing to the use of the automatic salivary segmentation algorithm; this is the main contribution of the current study. The learning period of a trainee for salivary segmentation may no longer be needed, leading to reliable assessment of salivary function (Fig. 4). The trainee generated some deviating results, whereas the automatic algorithm presented results comparable with the established reference data. Indeed, the automatic algorithm performed as well as the highly experienced humans who trained the CNN.

One more interesting point of the study is that radioactivity as represented by the %ID appears to be more reliable than the size of salivary glands as segmented by the CT, which was more prominent in the submandibular glands than the parotid glands (Table 1). The reason for this is not clear, but the greater Hounsfield unit of the submandibular glands may have influenced the operators to include more salivary tissue for the submandibular glands^39^, which might have led to more reliable inclusion of radioactivity. The automatic salivary segmentation algorithm sometimes rolled out segmentation results that appeared to be discrepant with human segmentation results. However, even in those cases, the %ID was not significantly different especially for the submandibular glands (Supplementary Fig. 5). Generally, the mismatches in VOI volume were not clinically significant in a majority of the cases of %ID, which has unique clinical implications for nuclear imaging studies different from external radiation therapy planning. Some minor mis-segmentation results would not seriously harm patient management, which provides more flexibility to the diagnostic application of our proposed algorithm.

In conclusion, with the aid of a CNN, we developed a quantitative salivary gland SPECT/CT protocol that is relevant for clinical applications without redundant radiation exposure.

## Limitations

This study has several limitations and drawbacks. The sample size for the performance comparison between the developed algorithm and the human beginner was only 20 cases, which might not be sufficient for a reliable comparison. However, the performance of the beginner tended to improve during the comparison process, resulting in an experienced beginner at the later time points. Therefore, it was difficult to verify the poor performance of the human trainee (Supplementary Table 2) because she was trained during the study and is currently well experienced. We sincerely attest that she was a genuine beginner at the time of comparison with the AI algorithm. In addition, the developed salivary segmentation algorithm was not validated using an external reference standard. However, because the developed algorithm is not only for salivary gland segmentation but also for quantitative SPECT data, there is no commercial software currently available for the validation of our algorithm.

## Materials and methods

### Dataset

Three datasets were used in the current study. The first was collected between August 2017 and September 2018 and used to assess the reproducibility of salivary segmentation by human experts (*n* = 30). The second set was recruited between September 2016 and November 2018 for the development of an automatic segmentation algorithm based on deep learning (*n* = 333). The third set was compiled between December 2018 and January 2019 to compare the performance of the developed automatic segmentation algorithm with that of a human beginner (*n* = 20) (Table 4). The three patient groups were indistinguishable by age and sex but differed in terms of their underlying disease (*p* < 0.0001 by a chi-square test). The patient demographics are available in the Supplementary Table 1. The original salivary gland SPECT/CT protocol consisted of SPECT/CT at 20 min, sialagogue stimulation (lemon powder 2 g, Lemona, Kyung Nam Pharm. Co., Korea), and then post-stimulatory SPECT/CT at 40 min after the injection of Tc-99m pertechnetate (555 MBq, Mo-99/ Tc-99m generator, Samyoung, Korea)^12^. The SPECT was obtained for 1 min in a continuous acquisition mode. We omitted the post-stimulatory 40-min CT to minimize CT-induced radiation exposure. In the first group, 19 patients underwent the original protocol and 11 patients underwent the new protocol (i.e., the original protocol without the 40-min CT) (Fig. 1a). Instead of the 40-min CT, the 20-min CT was employed to reconstruct the 40-min SPECT after misregistration correction using the vendor-provided software (Hybrid QC, Preparation for Q.Metrix, GE) (Fig. 1b). The second group experienced the modified protocol without the 40-min CT (Fig. 1a). The 20-min set of SPECT and CT was employed to train the automatic segmentation algorithm. The last group only underwent the proposed modified protocol (Fig. 1a). Details of the SPECT/CT acquisition and reconstruction parameters are provided in the “Supplementary material S1”. This study was retrospectively planned. The use of the salivary gland SPECT/CT data was approved by the institutional review board (IRB) and the acquisition of informed consent was waived by the IRB.

**Table 4:** Patient characteristics.

	For reproducibility (*n* = 30)	For development of automatic segmentation algorithm (*n* = 333)	For comparison with a human beginner (*n* = 20)	*P* value
Age^a^ (years)	52.27 ± 18.40	50.54 ± 15.04	49.25 ± 15.26	0.7728
Sex (male:female)	9:21	103:230	6:14	0.9912
**Underlying disease (cause of referral)**				
Dry mouth	*n* = 8	*n* = 118	*n* = 5	< 0.0001
Salivary gland tumor	*n* = 0	*n* = 41	*n* = 2	
Sialolithiasis	*n* = 3	*n* = 26	*n* = 5	
Post-RAI therapy for thyroid cancer	*n* = 5	*n* = 12	*n* = 0	
Pre-RAI therapy for thyroid cancer	*n* = 2	*n* = 75	*n* = 0	
Salivary gland operation	*n* = 10	*n* = 34	*n* = 5	
Others	*n* = 2	*n* = 27	*n* = 3	

^a^Age is mean ± standard deviation.

### Manual segmentation for quantitation of uptake and excretion

The uptake (%ID) of each salivary gland was calculated using the typical quantitative SPECT/CT approach (Q.Metrix, GE), which required the segmentation of each salivary gland. Manual segmentation is a time-consuming and laborious process because multiple regions of interest (ROI) drawings (~ 30 ROIs for a single parotid and ~ 20 ROIs for a single submandibular gland) were required to generate voxels of interest (VOI) for the corresponding salivary gland on the transaxial CT images^12^. We used a soft tissue CT window (level 40 and width 400) for salivary segmentation. The %IDs should be obtained for both 20-min and 40-min SPECTs to calculate the percent excretion fraction (%EF) for each salivary gland: the %EF was calculated as 100 × (20-min %ID–40-min %ID)/20-min %ID. The ROIs were drawn once every 2–3 slices, and the slices were interpolated on the Q.Metrix software. The entire process of calculating the %ID and %EF of the four salivary glands took at least 15 min per SPECT/CT scan (Supplementary Fig. 1).

### Reproducibility of salivary gland segmentation by humans

We calculated the reproducibility of manual salivary segmentation to investigate the competency of the human experts who contributed to the development of the automatic segmentation algorithm. The assessment was based on the 20-min set of SPECT and CT in 60 parotid and 56 submandibular glands, excluding four resected submandibular glands, from the first group of patients (*n* = 30) (Table 4 and the Supplementary Table 1). Two parameters were obtained by the human experts: VOI size (in ml) from the CT and %ID from the SPECT. For inter-operator reproducibility, two nuclear medicine physicians (JHK and DGO) independently manually segmented the salivary glands. For intra-operator reproducibility, another Ph.D. expert (JHH) conducted the segmentation process twice with a two-week waiting period in-between.

### Correction of misregistration between the 20-min CT and 40-min SPECT

We had to prove that we could omit the 40-min CT without the loss of information. This meant that the 40-min SPECT should be able to be reconstructed with the 20-min CT, which in turn leads to misregistration issues. We employed the vendor-provided quality control function of the reconstruction software (Hybrid QC, Preparation for Q.Metrix, GE) to align the 40-min SPECT with the 20-min CT (Fig. 1b). Nineteen of the 30 patients in the first group experienced the original salivary gland SPECT/CT protocol (complete set of SPECT/CT at both 20 min and 40 min post-stimulation)^12^. In the 38 parotid and 34 submandibular glands (excluding 4 total resected submandibular glands) of the 19 patients, the effects of misregistration correction were examined by one investigator (DGO). The %ID of 40-min SPECT was generated using the 20-min CT with or without misregistration correction and the effects of the correction were compared to the reference 40-min SPECT %ID obtained from the 40-min CT of the same time.

### Development of automatic segmentation algorithm for %ID

**Figure 5:**
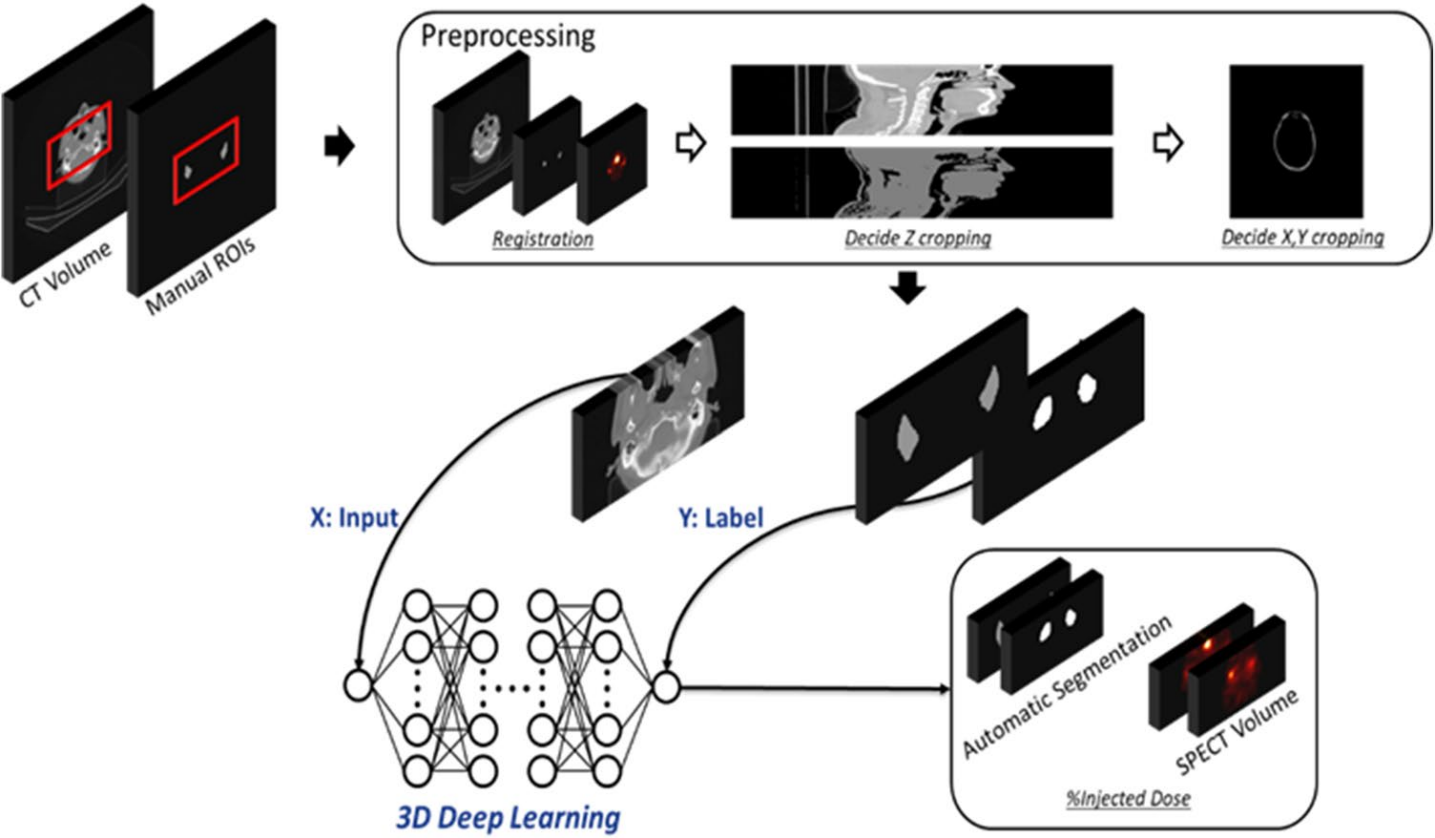
Schematic diagrams of the deep-learning-based salivary gland segmentation for %ID measurement using quantitative SPECT/CT.

We used the 20-min set of SPECT and CT from 333 patients (266 for training and 67 for testing) for network training and validation (Table 4 and the Supplementary Table 1). First, the three experts who had participated in the reproducibility analyses manually drew salivary ROIs for segmentation using the CT images from the Q.Metrix software, generating salivary volume data files (JHK 50 cases, DGO 142 cases, and JHH 141 cases). Second, the images were cropped into a 256 × 128 × 64 matrix to reduce the memory consumption during CNN training. The *z* cropping was based on the *z* profile of soft tissue and *x* and *y* cropping was performed with the maximum intensity projection images using entire CT volumes. Third, a deep convolutional neural network (CNN) algorithm was trained end-to-end using the CT as input and the result of the human-segmented salivary volume as label. Lastly, the automated segmented volume was applied to the 20-min SPECT, thereby generating the %ID of the salivary glands (Fig. 5). The architecture details of the deep CNN are noted in the literature^40^ and in Supplementary Fig. 2. The calculation processes for the %ID in the automated algorithm are also described in the “Supplementary material S1” and the literature^40^.

### Comparison of the developed automatic segmentation algorithm with a human beginner

The segmentation results of the developed algorithm in 20 patients who experienced the proposed salivary gland SPECT/CT (20-min SPECT/CT, sialagogue stimulation, and 40-min SPECT only) (Fig. 1a) from December 2018 to January 2019 were compared with those of a trainee (HGR). The trainee manually segmented the parotid and submandibular glands for the %ID at 20 min and 40 min post-stimulation, after which the %EF was calculated. The automatic segmentation results of the 20-min CT were not only applied to the 20-min SPECT (for 20-min %ID), but also to the 40-min SPECT (for 40-min %ID), leading to the %EF calculation. Here, we adopted data from 43 normal patients who had undergone salivary gland SPECT/CT in our previous report^12^. The reference data were acquired from patients with proven normal salivary gland function, whereas the current 20 patients had a variety of salivary gland diseases (Table 4 and the Supplementary Table 1). The principal investigator (WWL), with more than 25 years of experience in clinical nuclear medicine practice, determined the apparent normal salivary glands in a clinical context (i.e., no tumor, no operation, no salivary stone, no RAI therapy, and no proven sialadenitis) and visual assessment of the SPECT/CT (i.e., substantially high uptake and post-stress excretion). Thirty-six parotid and 32 submandibular glands were then selected to be apparent normal salivary glands from the 20 patients (Table 4).

### Statistical analysis

Parameter differences were analyzed using a paired t-test for two interrelated groups or an analysis of variance (ANOVA) test for three groups after confirming the variance equality using Levene’s test and the data normality using the Kolmogorov–Smirnov test. Else, nonparametric analyses such as the chi-square test or Kruskal–Wallis test were applied. Reproducibility was assessed using the intraclass correlation coefficient (ICC) of a two-way model (MedCal, version 12.4.0.0). The correction effects for the misregistration between the 20-minure CT and 40-minure SPECT were evaluated using a bias ± repeatability coefficient of 95% limits of agreement in a Bland–Altman analysis.

The DSC, measuring the overlap between manual and automated segmentation results, was calculated for the quantitative evaluation of the network according to the following equation:

$${\text{DSC}}({\text{P}},{\text{T}}) = \frac{{2 \times \left| {{\text{P}} \cap {\text{T}}} \right|}}{{\left| P \right| + \left| T \right|}}$$

where ***P ∩ T*** is the element-wise product of ***P*** and ***T***, which are the automatic and manual segmentations, respectively. Each layer was updated using error backpropagation with the Adam (adaptive moment estimation) optimizer, a stochastic optimization technique (x).

We also assessed the correlation and mean absolute percentage error for the parotid %ID and submandibular %ID using segmentation methods. We performed fivefold cross-validation to confirm the consistency of performance. A *p* value of less than 0.05 was considered statistically significant.

### Ethics approval

This retrospective data analysis study involving human participants was in accordance with the ethical standards of the institutional and national research committee and with the 1964 Helsinki Declaration and its later amendments or comparable ethical standards. The Human Investigation Committee (IRB) of Seoul National University Bundang Hospital approved this study. The IRB waived the acquisition of the informed consent. All the methods were carried out in accordance with relevant guidelines and regulations.

## Supplementary Information

Supplementary Information.
