# Supplementary material for: Quantitative salivary gland SPECT/CT using deep convolutional neural networks
Source: Sci Rep. 2021 Apr 9;11:7842. doi: 10.1038/s41598-021-87497-0 (PMC8035179; doi:10.1038/s41598-021-87497-0)
Supplement: Supplementary file 1 — Supplementary Information. [file 41598_2021_87497_MOESM1_ESM.docx]

**Quantitative Salivary Gland SPECT/CT using Deep Convolutional Neural Networks**

Junyoung Park; Jae Sung Lee; Dongkyu Oh; Hyun Gee Ryoo; Jeong Hee Han; and Won Woo Lee


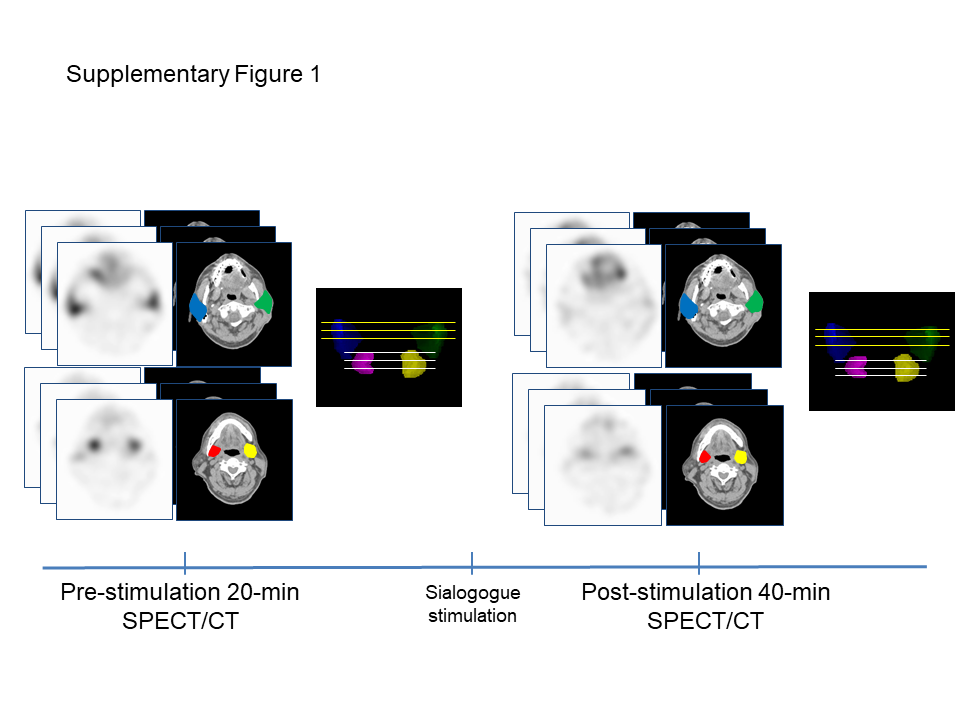


**Supplementary Figure 1**. The process of manual segmentation used for the parotid and submandibular glands before and after sialogogue stimulation. The uptake (%injected dose=%ID) of each salivary gland was obtained using quantitative SPECT/CT. The percent excretion fraction (%EF) for each salivary gland was calculated as 100 × (20-minute %ID – 40-minute %ID) / 20-minute %ID.


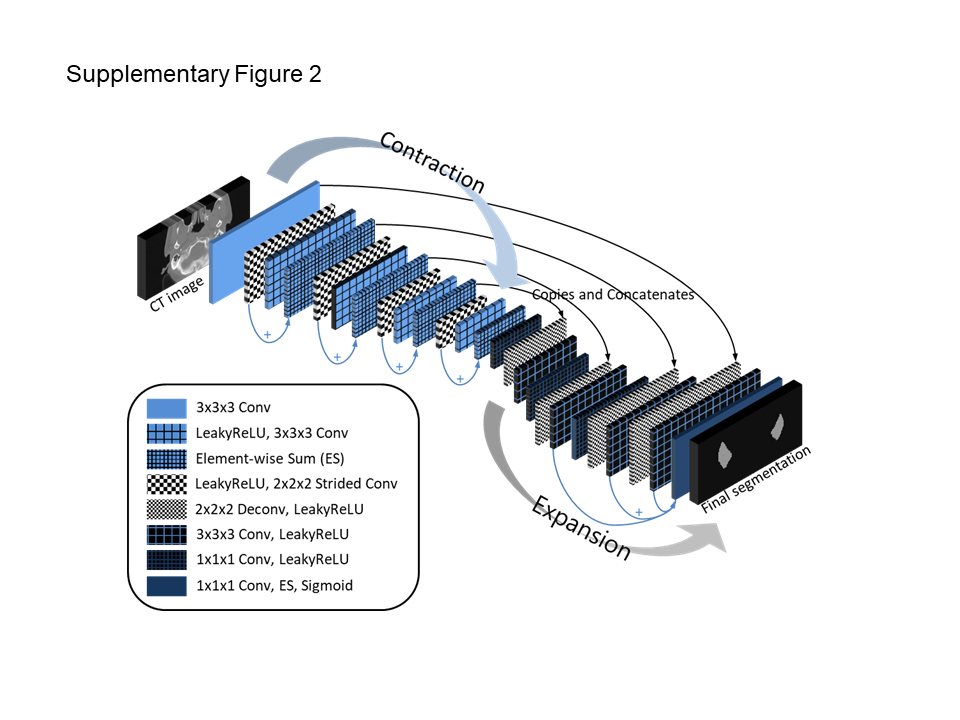


**Supplementary Figure 2.** Deep neural network architecture. The network learns an end-to-end mapping between CT and human-segmented salivary gland volume.

**
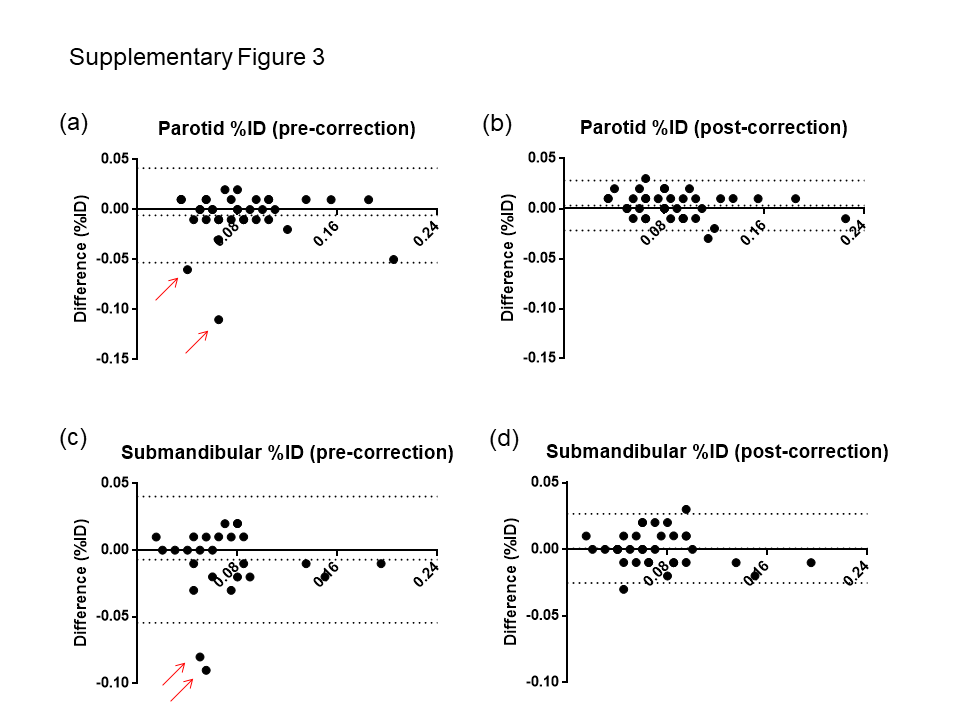
**

**Supplementary Figure 3**. Effects of mis-registration correction between 20-minunte CT and 40-minunte SPECT. The 40-minunte SPECT %IDs were calculated using the 20-minute CT (with/without correction) and the 40-minute CT. The X-axis means average %IDs of 20-minute CT and 40-minute CT, while the Y-axis denotes the absolute difference. (a) Parotid %IDs from the un-corrected 20-minute CT were significantly lower than those from the reference 40-minute CT in some cases (red arrows). (b) The correction procedure resulted in reduced bias (middle dashed line) and narrower 95% lines of agreement (LOA) (upper and lower dashed lines) as well as correction of the severely biased data. (c) Submandibular %IDs showed more bias, wider 95% LOA, and some seriously biased cases before the misregistration correction (red arrows). (d) The minimalized bias, narrowed LOA, and absence of severe discrepancy were clearly appreciated for the submandibular %IDs post-correction.

**
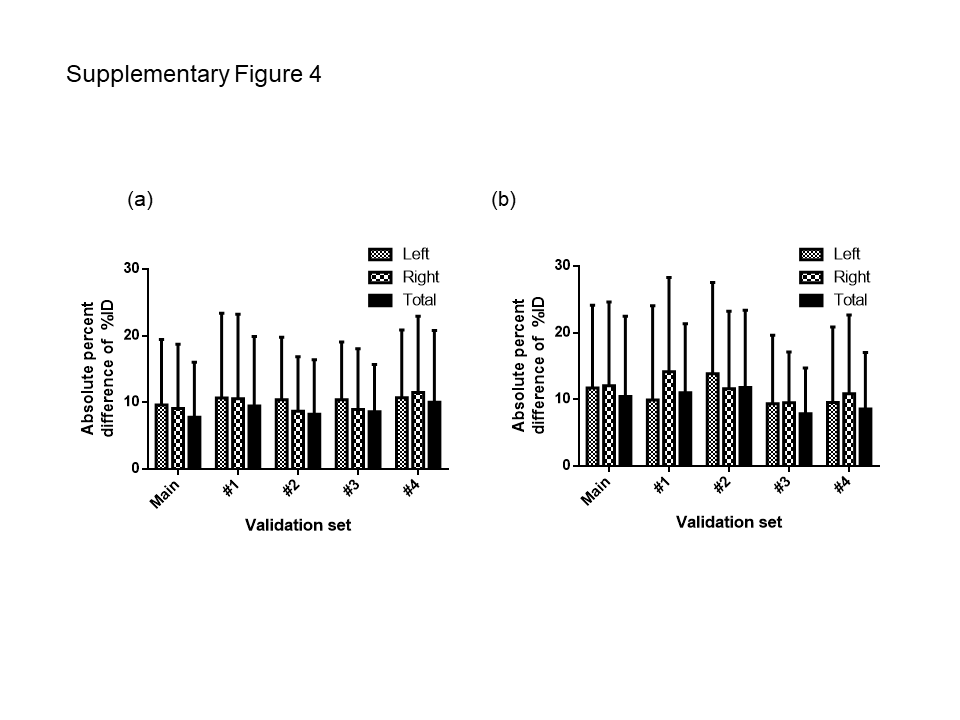
**

**Supplementary Figure 4**. Absolute percentage difference of %ID of parotid (a) and submandibular glands (b) between measurements using manual and deep-learning-generated volumes: results of five-fold cross-validation.


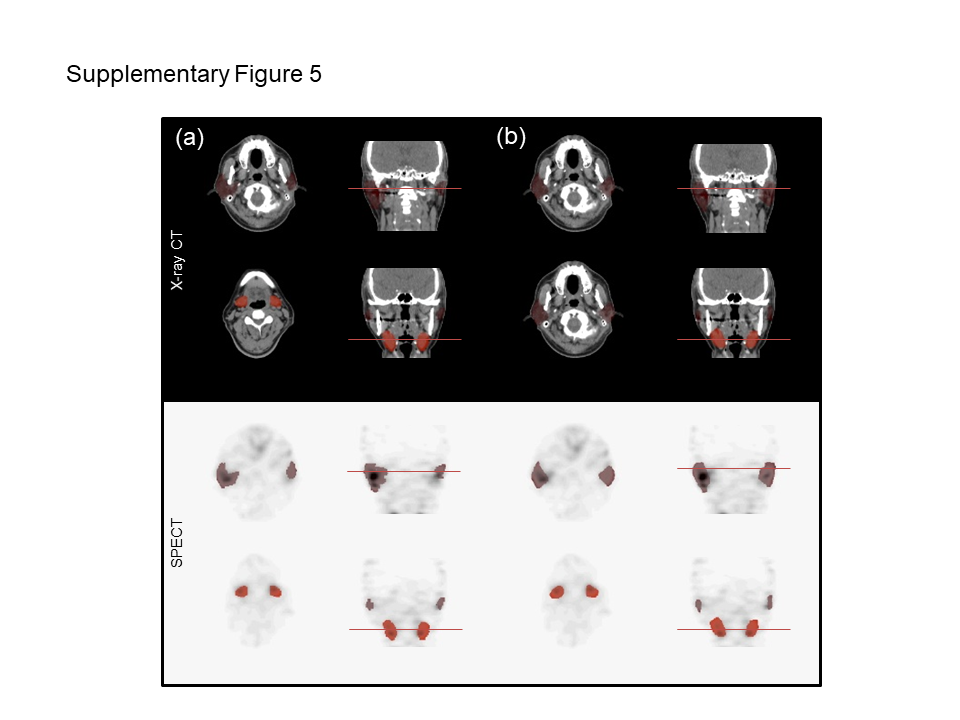


**Supplementary Figure 5.** Discrepant results of salivary gland segmentation of parotid glands but relatively consistent segmentation of submandibular glands. (A) Manually segmented ROI. (B) Deep-learning-generated automatic ROI.

**SUPPLEMENTARY MATERIAL**

**Supplementary Table 1. Details of the underlying diseases of the patients**

|  |  | For reproducibility (n=30) | For development of automatic segmentation algorithm (n=333) | For comparison with a human beginner (n=20) | P value |
| --- | --- | --- | --- | --- | --- |
| Underlying disease (cause of referral) | Dry mouth | n=8 | n=118 | n=5 | <0.0001 |
|  | Salivary gland tumor | n=0 | n=41 (Rt.SM=4, Lt.SM=2, Rt.P=16, Lt.P=19) | n=2 (Rt.P=1, Lt.SM=1) |  |
|  | Sialolithiasis | n=3 (Rt.SM=3) | n=26 (Rt.SM=12, Lt.SM=9, Rt.P=4, SMG duct=1) | n=5 (Rt.SM=4, Lt.SM=1) |  |
|  | Post-RAI therapy for thyroid cancer* | n=5 | n=12 | n=0 |  |
|  | Pre-RAI therapy for thyroid cancer | n=2 | n=75 | n=0 |  |
|  | Salivary gland operation | n=10 (Rt.P stone removal=1, Lt.P partial=5, Rt.SM total=2, Lt.SM total=2) | n= 34 (Rt. SM total=1, Rt.SM stone removal=3, Lt. SM stone removal=4, Rt.P partial=11, Lt.P partial=14, Lt.P total=1) | n=5 (Rt.P partial=1, Lt.P partial=2, Rt.SM total=1, Lt.SM stone removal=1) |  |
|  | Others | n=2 | n=27 | n=3 |  |

* Mean dose of radioactive iodine (RAI): 136.8±22.6 mCi (range: 100~154 mCi) and 114.2±48.3 mCi (range: 30~150 mCi) for the first and the second group, respectively. No significant difference (p=0.3389).

Rt.P=Right parotid gland; Lt.P=Left parotid gland; Rt.SM=Right submandibular gland and Lt.SM=Left submandibular gland.

**Supplementary Table 2. Dice similarity coefficient (DSC) values between the developed AI algorithm and the humans.**

|  |  | 1^st^ observer (n=12) | 2^nd^ observer (n=28) | 3^rd^ observer (n=27) | Trainee (n=20) |
| --- | --- | --- | --- | --- | --- |
| AI | Parotid | 0.74±0.06 | 0.76±0.05 | 0.74±0.07 | 0.76±0.05 |
|  | Submandibular | 0.72±0.12 | 0.74±0.07 | 0.71±0.15 | 0.76±0.07 |

Data is mean ± standard deviation

**Parameters for acquisition and reconstruction of the salivary gland SPECT/CT**

Patients had been fasting for at least 2 h before participating in the study. Tc-99m pertechnetate was eluted out of the Mo-99/Tc-99m generator (Samyoung, Korea) using 0.9% normal saline. The injected dose was 555 MBq as measured in the dose calibrator (CRC-15R, Capintec, USA); the calibrator was cross-calibrated with the SPECT/CT scanner (NMCT670, GE, USA). The cross-calibration factor or system sensitivity was determined using three times of phantom studies as 152.5 cpm/µCi. The following information was recorded to calculate the quantitative parameter (%ID): radioactivity and its measurement time before and after injection, injection time of the patients, and imaging start time. At 20- and 40-minute post-injection of Tc-99m pertechnetate, SPECTs were acquired for 1 min with the patients lying on the scanner table. Planar images were not obtained. The SPECT acquisition parameters were as follows: low-energy high-resolution collimation, peak energy of 140 keV with 20% windowing (126~154 keV), scatter energy of 120 keV with 10% windowing (115~125 keV), continuous mode acquisition for 1 min without body contour option, counter-clockwise rotation and zoom factor of 1.5. The SPECT images were reconstructed using the iterative ordered subset expectation-maximization algorithm (2 iterations and ten subsets). During reconstruction, triple corrections (CT attenuation correction, scatter correction, and resolution recovery) were employed. After applying a post-reconstruction filter (Butterworth; frequency of 0.48 and order of 10), the SPECT images were generated in a matrix of 128 × 128 and slice thickness of 2.95 mm.

Immediately after SPECT acquisition, helical CT was performed using the following parameters: tube voltage: 120 kVp, tube current: 30 mA, detector collimation: 16×1.25 = 20 mm, helical thickness: 2.5 mm, table speed: 37 mm/s, table feed per rotation: 18.75 mm/rot, tube rotation time: 0.5 s, pitch: 0.938:1, matrix: 512×512, slice thickness: 2.5 mm at the transaxial plane, and no increment. All the image processing was conducted using the vendor-provided workstation (Xeleris 3.1, GE).

**Neural network architecture**

A modified 3D U-Net is used to learn end-to-end between the CT and human-segmented salivary volumes. We used an element-wise sum array to forward feature map from one stage of the network to the other. **Supplementary Figure 1** illustrates the network architecture. The network comprises the analysis and synthesis paths, such as the conventional U-Net (X). For the analysis path, each layer comprises a 3 × 3 × 3 convolution, each followed by a leaky rectified linear unit (leaky ReLU) as an activation function and a 2 × 2 × 2 strided convolution for down-sampling. For the synthesis path, each layer comprises a 3 × 3 × 3 convolution, each followed by a leaky ReLU 1 × 1 × 1 convolution, and 2 × 2 × 2 de-convolution for up-sampling. The leaky ReLU allows a small gradient when the unit is inactive. Compared to the conventional U-Net, we adapted an element-wise sum array for both the paths to forward feature map from one state of the network to the other. It is applied between the output of a 2 × 2 × 2 strided convolution and a 3 × 3 × 3 convolution to forward feature map for the analysis path and is placed before the Sigmoid activation function to add the 3 × 3 × 3 convolution results of the previous three layers for the synthesis path.

We also employed batch normalization for better training performance and symmetric skip connection to insert the local details of the feature maps from the analysis path to the synthesis path. The hyper-parameters used are as follows: 0.00005 is the initial learning rate, which is reduced by half if there is no improvement after ten epochs; 172 iterations per each epoch. The training time was roughly 10 min/epoch, and the number of total epochs was 80 when i7-7700K CPU and one GTX 1080 TI GPU were used.

**Calculation of %ID from the automatic segmentation algorithm**

We calculated %ID by applying the manual and automatic VOIs to the quantitative SPECT images. First, the total injected radioactivity was calculated using the following equation:

$A_{inj}\left( \mathrm{Total} \right)=A_{pre}\times e^{\left( -\ln\left( 2 \right)\times\frac{T_{inj}-T_{pre}}{T_{1/2}} \right)}-A_{post}\times e^{\left( \ln\left( 2 \right)\times\frac{T_{post}-T_{inj}}{T_{1/2}} \right)}$

where *A_pre_* and *A_post_* are, respectively, the pre-injection and post-injection (residual) activities; *T_pre_* and *T_post_* are, respectively, the measurement times for these activities; *T_inj_* is the injection time; and *T*_1/2_ is the half-life of Tc-99m.

Subsequently, the activity and %ID in each salivary gland was calculated using the following equations:

$$A_{inj}\left( Salivary \right)=R_{cnt}/S\times e^{\left( \ln\left( 2 \right)\times\frac{T_{scan}-T_{inj}}{T_{1/2}} \right)}$$

$$\%ID=A_{inj}\left( Salivary \right)/A_{inj}(Total)\times100$$

where *T_scan_* is the scan time, *S* the system sensitivity, and R*_cnt_* the calculated count from the segmented area of the SPECT image.

**Results of the mis-registration correction between the 20-minute CT and the 40-minute SPECT**

The correction of the mis-registration between the 20-minute CT and the 40-minute SPECT was performed by the second investigator (DGO). SPECT was re-positioned and adjusted to the CT, and thus, the VOI sizes of the glands from the fixed CT images did not significantly differ among the 20-minute CT (before and after correction) and the 40-minute CT: parotid VOI (26.35±11.55 mL vs. 27.14±11.11 mL vs. 25.93±11.33 mL, p=0.896 by ANOVA test) and submandibular VOI (11.05±3.50 mL vs. 11.52±3.44 mL vs. 10.52±3.74 mL, p=0.512 by ANOVA test). Only the %ID was used as an indicator for misregistration correction.

The 40-minute SPECT %IDs were separately reconstructed using the 20-minute CT with/without the misregistration correction and the reference 40-minute CT. In fact, the %IDs were not significantly different among the three conditions: parotid %ID (0.09±0.04% vs. 0.08±0.04% vs. 0.09±0.04%, p=0.614 by ANOVA test) and submandibular %ID (0.07±0.04% vs. 0.06±0.04% vs. 0.07±0.04%, p=0.638 by ANOVA test). However, in Bland-Altman analyses, the effects of the misregistration correction were clearly noted: bias ± repeatability coefficients (half-width of the 95% LOA) for %IDs before correction (-0.0058±0.0473% for parotid and -0.0071±0.0474% for submandibular gland) significantly improved (0.0032±0.0250% for parotid and 0.0006±0.0260% for submandibular gland) after the correction (**Supplementary Figure 2**), which suggested that the 40-minute SPECT was able to stand alone without accompanying CT acquisition.
